# *N*-Acetylglucosamine: Production and Applications

**DOI:** 10.3390/md8092493

**Published:** 2010-09-15

**Authors:** Jeen-Kuan Chen, Chia-Rui Shen, Chao-Lin Liu

**Affiliations:** 1 Department of Environment and Biotechnology, Refining & Manufacturing Research Institute, CPC Corporation, 217 Min-Sheng S. Rd, Chiayi, Taiwan; E-Mail: 078450@cpc.com.tw (J.-K.C.); 2 Department of Medical Biotechnology and Laboratory Science, Chang Gung University, Kweishan, Taoyuan, 259 Wen-Hwa 1st Road, Kweishan, Taoyuan, Taiwan; E-Mail: crshen@mail.cgu.edu.tw (C.-R.S.); 3 Graduate School of Biochemical Engineering and Department of Chemical Engineering, Ming Chi University of Technology, Taishan, Taipei, 84 Gung-Juan Road, Taishan, Taipei, Taiwan

**Keywords:** N-acetylglucosamine, chitin, carbohydrate

## Abstract

*N*-Acetylglucosamine (GlcNAc) is a monosaccharide that usually polymerizes linearly through (1,4)-β-linkages. GlcNAc is the monomeric unit of the polymer chitin, the second most abundant carbohydrate after cellulose. In addition to serving as a component of this homogeneous polysaccharide, GlcNAc is also a basic component of hyaluronic acid and keratin sulfate on the cell surface. In this review, we discuss the industrial production of GlcNAc, using chitin as a substrate, by chemical, enzymatic and biotransformation methods. Also, newly developed methods to obtain GlcNAc using glucose as a substrate in genetically modified microorganisms are introduced. Moreover, GlcNAc has generated interest not only as an underutilized resource but also as a new functional material with high potential in various fields. Here we also take a closer look at the current applications of GlcNAc, and several new and cutting edge approaches in this fascinating area are thoroughly discussed.

## 1. Introduction

Carbohydrates, with the general formula C_m_(H_2_O)_n_, are the most abundant organic compounds in living organisms. Also known as saccharides, carbohydrates are involved in a variety of biological events. Fox example, they serve as the main source and storage form of energy as well as structural components. Monosaccharides, the monomers of carbohydrates, serve as the main source of energy for metabolism (e.g., glucose) and are used in biosynthesis [1]. Their derivatives are also important components of coenzymes and the backbone of genetic molecules. Furthermore, carbohydrates are essential in cell interactions [2], signal transduction [3,4], antibody recognition [5], tumor metastasis [6], hormones [7] and musculoskeletal physiology [8,9].

*N*-Acetylglucosamine (GlcNAc), 2-acetamino-2-deoxy-β-d-glucose or 2-(acetylamino)-2-deoxy-d-glucose, is a monosaccharide derivative of glucose and is widely distributed worldwide. The molecular formula of this amino monosaccharide is C_8_H_15_NO_6_, and its molecular weight is 221.21. In general, it is a white and slightly sweet powder that melts at 221 °C. The solubility of GlcNAc is 25% in water, and 1% aqueous solutions are colorless and clear.

GlcNAc polymerizes linearly with (1,4)-β-linkages and is the monomeric unit of the polymer chitin, the second most abundant carbohydrate after cellulose. Chitin is present in arachnids, most fungal cell walls, insect exoskeletons, the shells of crustaceans and parts of invertebrates [10,11]. It is also present as an extracellular polymer of some microbes [12,13]. Isolated chitin exists as two allomorphs, namely α-chitin and β-chitin. α-chitin is the most abundant by far and is present in shrimp and crab shells. The rarer β-chitin is found in squid pens and is commercially more expensive. To date, it is not possible to obtain β-chitin either from solution or by *in vitro* biosynthesis.

In addition to being a structural component of homogeneous polysaccharides like chitin, GlcNAc is also a constituent of heterogeneous polysaccharides, such as murein [10–14] and hyaluronic acid (also called hyaluronan or hyaluronate, HA) [14]. Murein is the basic component of the bacterial cell wall and consists of crosslinked peptide chains with repeating GlcNAc and muramic acid residues [14]. HA is a linear heteropolysaccharide that is composed of repeating d-glucuronic acid and GlcNAc residues [14]. As early as 1943, HA was discovered in bovine eyes, and its defect causes disease. In fact, it is the major component of the extracellular matrix and is extensively distributed in connective, epithelial and neural tissues [15–20]. HA performs numerous roles in cell motility, inflammation and cancer metastasis [21]. In addition to its contributions in eukaryotes, HA is also a component of cell coatings in some bacteria [22]. HA can be produced by biochemical engineering techniques [23,24].

Chondroitin is a glycosaminoglycan that is composed of the same repeats as HA but is sulfated at position C-4 or C-6 of the *N*-acetylgalactosamine residues. It is also plentiful in connective tissues, particularly in blood vessels, bone and cartilage. In fact, GlcNAc and sulfated GlcNAc can be isolated from heparin or keratin sulfate [25,26]. Heparin, which is produced by basophils and mast cells, consists of a variably-sulfated repeating disaccharide unit and acts as an anticoagulant. The major repeating disaccharide unit of keratin sulfate, also called keratosulfate, is composed of galactose and sulfated GlcNAc. Keratin sulfate is distributed in the cornea, cartilage and bone and usually acts as a cushion in joints to absorb mechanical shock.

Moreover, GlcNAc molecules with various functional group substitutions serve as ingredients of cell surface decoration and/or are directly involved in cell interactions [5]. Indeed, these carbohydrates may increase the specificity and interaction force between cells. For example, blood type usually depends on the oligosaccharides present of the surface of erythrocytes in human beings, and GlcNAc appears to be the basic element of the H antigen in ABO blood grouping [5]. GlcNAc was also identified in the two epitopes Sialy-Lewis X and Sialy-Lewis A. They are ligands that capture selectins for adhesion, but they also contribute significantly to metastasis [6,27–29].

In addition, glycoproteins that contain GlcNAc can be found in the mucous membranes of the digestive tract. However, saccharide modification may promote pathogen binding. For example, GlcNAc-β-(1–4)-GlcNAc is present in membrane proteins of brain microvascular endothelial cells (BMECs) and acts as the ligand for *Escherichia coli* K1 outer membrane protein A (OmpA) to mediate bacterial transport through the blood-brain barrier in neonates [30,31].

Generally, oligosaccharide modification is proposed to play a role in structural stability and interaction with other molecules. In particular, *O*-GlcNAcylation affects performance, including activity, stability and/or localization [32]. *O*-linked GlcNAc usually modifies nuclear pore proteins that are involved in nuclear pore assembly and function [33]. However, *N*-GlcNAcylated glycoproteins are cleared more rapidly by lysosomal glycosidases [34].

In plants, GlcNAc has been found in bromelain, ricin agglutinin and *abrus* agglutinin [7,35–37]. In humans, GlcNAc is frequently observed in glycoproteins, such as tissue plasminogen activator [38]. It is also detected in mammalian growth factors and hormones [35], including follicle-stimulating hormone (FSH), luteinizing hormone (LH), human menopausal gonadotropin (hMP), pregnant mare serum gonadotropin, thyroid-stimulation hormone (TSH) and human chorionic gonadotropin (hCG).

Recently, GlcNAc and its derivates been utilized in dietary supplements and for therapeutic development [8,39–41] due to its unique characteristics. Toxicity tests reveal that GlcNAc is non-toxic, supporting the essential safety issue, and the half-life of GlcNAc with 20 g intravenous injection is 220 min [42]. While 800 mg administrated *per os*, the mean maximum concentration (C_max_) of GlcNAc in plasma was 162.7 ± 125.2 ng/mL and the time for maximum concentration (T_max_) was 1.56 ± 1.23 h [43]. It was further demonstrated that 54% of the administered glucosamine (GlcN) was excreted into the urine in one day, indicating the clearance pathway of GlcNAc [42]. Therefore, GlcNAc can potentially be utilized in many significant applications.

## 2. Production of *N*-Acetyl-d-glucosamine

**Figure 1:**
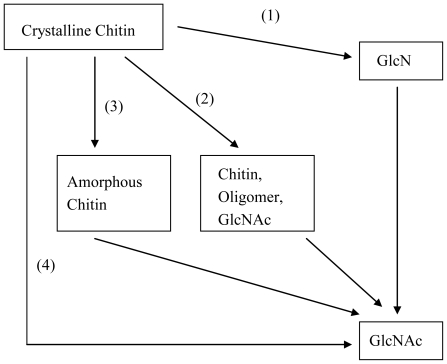
Production of GlcNAc using chitin as a substrate. (**1**) Chemical methods; (**2**) Enzymatic methods; (**3**) Improved enzymatic methods; and (**4**) Biotransformation methods.

In mammals, GlcNAc and GlcN are components of glycoproteins, proteoglycans, glycosaminoglycans (GAGs) and other connective tissue building blocks [15–20]. Despite being a building block of biomacromolecules, GlcNAc seldom exists in free form, except in human milk (at 600–1500 mg/mL) [44,45]. Because GlcNAc not only play a role in plant organogenesis and invertebrate embryogenesis [46] but also has therapeutic potential in the treatment of a wide variety of diseases, economic feed-stocks and high efficiency processes for producing GlcNAc are urgently needed in industry. Every year, about 100 billion tons of chitin are produced in nature [47–49], making chitin a suitable biomass resource for the production of GlcNAc. Using chitin as feed-stock, GlcNAc can be prepared through a process that is based on chitin hydrolysis. Figure 1 summarizes the processes of GlcNAc production to date.

### 2.1. Chemical methods to produce GlcNAc

GlcNAc has historically been commercially prepared by several companies via a process based on the acid hydrolysis of crude chitin. Generally, the chemical degradation of chitin can be performed through hydrolysis using a strong acid, such as HCl. The processing temperature and concentration of the acid should be carefully selected. These parameters must be high enough to sufficiently degrade chitin but not so high that the GlcNAc product is destroyed. Suitable reaction conditions include 15–36% HCl and about 40–80 °C. In this procedure, up to 6.42 g/L GlcNAc can be produced in 1 h [50]. However, there appears to be several problems in producing GlcNAc by the direct acid hydrolysis of chitin, including high cost, low yield (below 65%) and acidic waste created by the use of HCl [51].

An alternative process involves the use of GlcN. Chitin can be dissolved in concentrated HCl and heated in boiling water for 3 h. In such drastic conditions, the acetyl group of GlcNAc is removed. An additional *N*-acetylation reaction must then be performed to produce GlcNAc. In a typical preparation, GlcN is dissolved in 10% methanol and is *N*-acetylated with acetic anhydride in the presence of Dowex 1 (carbonate form). After filtration and crystallization, this process can generate an overall yield of 43% GlcNAc [52,53]. Another improved *N*-acetylation procedure was described by Zhan (2007). GlcN is *N*-acetylated using pyridine as a solvent in the presence of tributylamine and acetic anhydride. After a series of purification procedures, ~99% pure GlcNAc can be obtained. The yield of this method has been reported to be higher than 70% [54].

Although the production of GlcNAc by chemical methods is estimated to be sufficiently economic, the product is not considered a natural material due to its chemical modification [51]. In addition to *N*-acetylated products, trace amounts of *O*-acetylated and di-acetylated products have also been detected by LC-MS. Furthermore, solvent and tributylamine are present in the product, as determined by GC-MS (unpublished data). Thus, chemically produced GlcNAc usually tastes bitter due to these residual substances.

Several efforts have been made to improve the purity of GlcNAc produced by chemical methods. Ryosuke *et al.* (2000) combined the acid hydrolysis of chitin and ion-exchange membrane electrophoresis to prepare natural GlcNAc [55]. Zhan (2007) established a novel purification and recrystallization procedure to prepare GlcNAc with purity that was higher than 99.95% [54]. In addition, the ozone treatment has been developed [56]. Thus, it will be possible to use chemically modified GlcNAc as an inexpensive resource in food additives, cosmetics and pharmaceuticals in the future.

### 2.2. Enzymatic methods to produce GlcNAc

GlcNAc produced by chemical methods is not widely commercialized, not only due to technical reasons but also because of environment concerns. The large quantities of chemical waste resulting from chemical processes are not environmentally friendly. However, the enzymatic hydrolysis of chitin can produce GlcNAc under mild conditions.

The enzymes degrading chitin, chitinases, distributes in various organisms [10]. They involves in the physiological and pathological functions [57–62]. The ensemble of chitinolytic enzymes can contain endochitinases (EC 3.2.1.14), exochitinases (EC 3.2.1.52), chitobiosidases (EC 3.2.1.30) and *N*-acetylglucosaminidases (NAGases) (EC 3.2.1.96) that are produced and secreted from either a prokaryotic or eukaryotic organism [10]. Usually, the endochitinases within microbes cleave chitin randomly at internal sites on the glycosidic bonds between GlcNAc residues, and such generates the soluble and low molecular weight oligosaccharides (e.g., tetramers, trimers and dimers). To increase the solubilization of chitin is essential as the first step. Then, the exochitinases or chitobiosidases in some organisms catalyze the progressive release of dimers, starting at the non-reducing end of chitin. Finally, NAGases cleave the oligomers and dimers, which are produced by endochitinases and exochitinases, to generate GlcNAc [63,64]. Several crude enzymes isolated from *Trichoderma viride*, *Aspergillus niger*, *Carcica papaya* L. and *Aeromonium* have been found to degrade β-chitin and to produce GlcNAc efficiently [51]. In contrast, some other microbes, such as *Trichoderma hamatum* AB10282 strain (FERM BP-10623) or *Trichoderma harzianum* AB10283 strain (FERM BP-10624), produce GlcNAc in the medium without the presence of chitin or chitin derivatives [65].

For the industrial production of GlcNAc, a large quantity of enzymes can be purified from the mass production of microbes or genetically engineered microorganisms [66–68]. However, owing to the low physiological content of chitinase in organisms, the direct purification of chitinase, even from the fermentation broth of genetically engineered microbes, is expensive. Some commercial, unspecific, crude enzymes, such as cellulose, lysozyme, papain and lipase, also degrade chitin due to the presence of endo- and exochitinase in crude enzyme preparations [69–71]. These unusual enzymes are inexpensive, and large amounts of them can be easily obtained commercially.

Several enzymatic processes can be performed to produce GlcNAc. Sashiwa *et al.* (2002) described the effective production of GlcNAc from flake type α-chitin by crude enzymes derived from *Aeromonas hydrophila* H2330 [72]. In this study, the selective production of GlcNAc from α-chitin was achieved with a yield up to 77% in 10 days. Although high yield and purity was achieved, low productivity was the crucial defect of this method. Another process has been described by Pichyangkura *et al.* (2002) in which crude chitinases from *Bacillus licheniformis* SK-1 were used to digest α-chitin powder for the production of GlcNAc. However, only a 41% yield of GlcNAc was achieved along with the production of chitobiose [73].

Poor yields were reported in these studies using α-chitin as a substrate. The major impediment of the enzymatic hydrolysis process is the extremely low susceptibility of natural α-chitin due to its high crystallinity. In another experiment, the hydrolysis of β-chitin, which shows good swelling properties compared with α-chitin, was accomplished by crude enzymes (e.g., cellulose T and A), and a yield of nearly 100% was achieved [74]. It is worth noting that the amorphous portion of chitin is easily hydrolyzed, and the remaining tightly packed chitin is hydrolyzed slowly.

Almost 100% pure GlcNAc was produced using crude enzymes from *A. hydrophila* H2330 [72], while chitobiose was synergistically produced using *Bacillus* chitinases [73]. This finding indicates that endochitinase, exochitinase and NAGase are necessary for the complete digestion of chitin, and higher NAGase activity results in a higher purity of GlcNAc. Thus, the chitin crystalline structure and the enzymatic composition of the reaction are two crucial factors in the production of GlcNAc through enzymatic methods.

### 2.3. Improved enzymatic methods to produce GlcNAc

The ability of chitin to act as a substrate for enzymatic hydrolysis might be improved by treatments that lead to its decrystallization. Several productive processes have been used to improve the purity and productivity of GlcNAc. Most of these processes focus on the modification of the chitin crystal structure because decrystallized chitins are more susceptible to enzymatic hydrolysis than natural chitin.

The crystallographic parameters of chitin reveal that there are two anti-parallel molecules per unit cell in α-chitin, while only one is present in β-chitin. The proposed crystal structures of α-chitin and β-chitin are organized into sheets that are tightly held by a number of intra-sheet hydrogen bonds. In addition, some inter-sheet hydrogen bonds are also present in α-chitin but not in β-chitin. Thus, α-chitin is thermodynamically more stable than β-chitin [75]. When β-chitin crystals are destroyed during acid swelling, new crystals of α-chitin are produced during recrystallization. The crystallinity of different chitin substances can be detected by X-ray diffractograms [76]. The limited enzymatic hydrolysis of natural chitin may be attributed to a limited accessibility of β-glycosidic bonds in the interior of the chitin crystal structure. Thus, β-chitin is degraded much more readily than α-chitin due to its weak intermolecular forces [77]. Sashiwa *et al.* (2001) obtained GlcNAc from 1% β-chitin by hydrolysis with various commercial enzymes, such as cellulose, hemicellulase, papain, lipase and pectinase. The highest yield (~76%) was obtained after eight days of hydrolysis with 20 mg/mL of cellulose [51].

Heat-treating chitin in a hydrophobic solvent or surfactant is suggested to weaken the hydrogen bonding or hydrophobic interactions of the crystal structure and, thus, facilitate the action of enzymes for decomposition. Chitin colloidized is better access for enzymes digestion [78]. Kawasaki *et al.* treated chitin using straight-chain hydrocarbons, surfactants (e.g., Triton X-100 or Tween 20) and urea to loosen the chitin structure and then heated or ultrasonicated the mixtures. These treatments increase the conversion ratio of chitin [79].

It is also possible to efficiently decompose chitin by exposing it to compounds that destroy its crystal structure. However, hydrogen bonding and hydrophobic interactions are generally necessary to maintain the three-dimensional structure of enzymes as well. Thus, chitin-degrading enzymes may be inactivated under these strict conditions. Enzyme reactions must be performed under conditions that their catalytic activities remain, such as with low concentrations of chemical agents. Recently, some novel sodium dodecyl sulfate (SDS)-resistant endochitinases and thermostable chitinases were found in the fermentation broth of bacteria [80–82]. These enzymes could be applied to improve the hydrolytic efficiency of chitin in the future.

The most common source of chitin for producing GlcNAc is shellfish biomass. However, the supply of shrimp and crab shells is highly dependent on seasonal and environmental factors, leading to unpredictable limitations on production capacity. Another option to stabilize the chitin source for the production of GlcNAc is to utilize a non-shellfish chitin source. In several typical fungal species, at least 15% of chitin exists in their cell walls. Chen *et al.* established a method to produce chitin by culturing an *Actinomucor taiwanensis* fungus. The highest yield of chitin was achieved at 1.5 g/L using their fermentation method [83]. The crystal structure of fungal chitin has not yet been characterized, although it is hypothesized to be highly susceptible to enzymatic degradation.

GlcNAc can be produced using fungal biomass as a substrate. Bohlman *et al.* (2004) obtained GlcNAc of high purity by degrading fungal chitin using either acid or enzymes. The concentration of GlcNAc obtained by acid degradation was 6.42 mg/mL, while that obtained by enzymatic treatment was 4.04 mg/mL [50,84]. Raetz *et al.* (1998) compared several chitins from different sources and tested their susceptibility to enzymes isolated from *Penicillium janthinellum* P9. Their results also confirm that fungal chitin has a higher susceptibility to hydrolysis than arthropod chitin [85].

A continuous production system used to obtain GlcNAc from chitin was described by Louise *et al*. This system involves five essential unit operations: (1) the fermentation unit, which is required for both cell culture and the production of chitinolytic enzymes; (2) the enzyme recovery unit; (3) the pretreatment of the chitin substrate; (4) the two-stage chitin-hydrolysis reactor; and (5) the final purification unit to obtain GlcNAc [86].

In the first unit, *Serratia marcescens* QM B1466 is used to produce chitinolytic enzyme ensembles. After induction for four to six days, the chitinase activity in the medium exceeds 105 U/mL, and the NAGase activity in the medium is two-fold higher than that of chitinase. In the second unit, chitinolytic enzyme ensembles are concentrated using colloidal chitin adsorption or ultrafiltration. In the third unit, mechanical methods, such as ball milling and hammer milling, are used to increase the surface area of chitin. Physical-chemical methods, such as autoclaving and steam exploding, are then used to swell the chitin particles, and treatment with dimethyl acetimide and weak cosolvents are explored to disrupt the chitin inter-chain hydrogen bonds.

**Figure 2:**
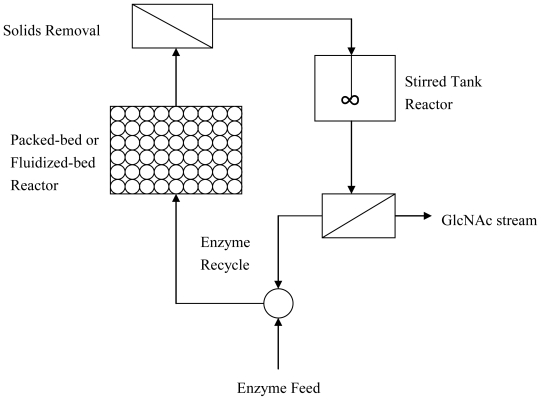
Two-stage chitin-hydrolyss bioreactor.

In the fourth unit, a novel two-stage chitin-hydrolysis bioreactor was designed to maintain a high rate of chitin hydrolysis and GlcNAc production. The configuration of the bioreactor is illustrated in Figure 2. Enzyme ensembles are pumped into the first reactor, which contains pretreated chitin particles as a packed bed or fluidized bed. Chitinases are adsorbed onto chitin beads, and the beads are steadily hydrolyzed. NAGase, which is not adsorbed onto the chitin beads, and the synergetic soluble fractions are released into the second stirred reactor. The solid content is recycled back to the first reactor. The soluble fractions are completely hydrolyzed by NAGase, making GlcNAc the sugar product of the two-stage reactor. The enzyme catalyst stream is continuously recycled into the reactor after removing GlcNAc by cross-flow filtration.

In the fifth unit, a continuous cross-flow ultrafiltration cell is used to separate the GlcNAc product from the chitinolytic enzyme ensemble. GlcNAc, with a purity greater than 98%, is recovered in the filtrate, and no protein contaminants are detectable. However, the primary oligosaccharide contaminant in the product is chitobiose, as detected by HPLC. Further purification procedures are necessary to recover GlcNAc from the filtrate.

This continuous production system is capable of producing GlcNAc at an average productivity of 78 g/L/h. The two-stage bioreactors are operated continuously over a 10-day period, reaching steady-state production levels after 28 h, and this system is estimated to be an economic process for the production of GlcNAc.

### 2.4. Production of GlcNAc by biotransformation

Although economic processes to produce GlcNAc by enzymatic hydrolysis have been established, the necessity to arrange so many units is a limiting factor. Efforts to screen indigenous microorganisms from the soil for their ability to degrade chitin have been performed. Li *et al*. (2005) isolated a microbe, which was identified as *Aeromonas caviae* DYU-BT4, from a soil sample in Taiwan. In a fermentation test using chitin particles as a substrate, high chitinase activity (588.6 U/L) was detected, and the concentration of reducing sugar in the medium was 0.88 g/L. The major substance in the hydrolysate of chitin was GlcNAc. After optimization of the fermentation process, about 7.8 g/L of GlcNAc was produced using 2% of colloidal chitin as a substrate. This result indicates that it is possible to produce GlcNAc using whole microbes [87].

A novel chitin-degrading microbe named *Chitinibacter tainanensis* was isolated from a soil sample that was collected in southern Taiwan and was proven to produce GlcNAc [88]. The GlcNAc yield was 75% using α-chitin as a substrate and greater than 98% using β-chitin as a substrate. A needle-like crystal was produced after the broth was concentrated and crystallized, and the purity of the product was determined to be greater than 99%. It is suggested that some factors, called chitin-degrading factors (CDFs), can hydrolyze chitin and are released during the proliferation of *C. tainanensis* in the presence of chitin, with the concomitant production of acidic metabolite. Interestingly, the microbe is killed due to the acidic condition of the medium. However, CDFs still hydrolyze chitin, and the final product, GlcNAc, accumulates in the medium without being consumed by the microbe because it is dead [89].

**Figure 3:**
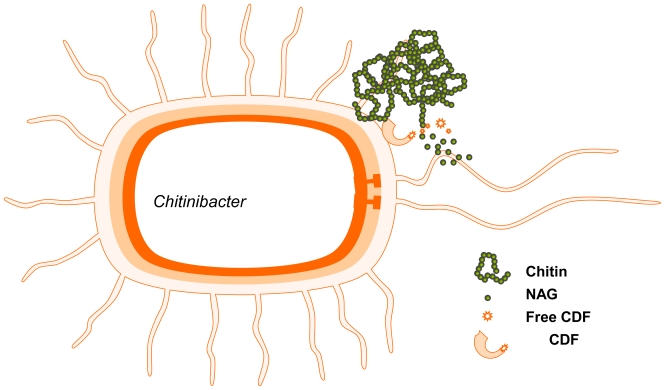
Production of GlcNAc by biotransformation.

Further studies indicate that CDFs are located on the surface of *C. tainanensis*. Thus, even if the microbes die, CDFs are still docked on the bacterial debris and maintain their chitinolytic activity. To determine how CDFs hydrolyze chitin, their endochitinase and NAGase activities have been measured. Both of these enzyme activities are found in live bacteria and in dead cell debris. The specific activity of NAGase is much higher than that of endochitinase. Therefore, GlcNAc is the only product of this biotransformation method. The mechanism of CDFs is similar to that of cellulosome from mesophilic bacteria [90] and is illustrated in Figure 3.

Another way to produce GlcNAc is through the genetic modification of microorganisms using glucose as a substrate. Metabolic pathways for GlcN and GlcNAc synthesis can be modified by genetic engineering techniques to increase enzyme activities, overexpress proteins, reduce product inhibition and increase the affinity of the substrate. A genetically engineered *E. coli* strain has been developed by Den *et al.* [91] in which GlcN-6-P acetyltransferase, GlcN-6-P synthase and GlcN-1-P acetyltransferase are overexpressed, while GlcN-6-P and GlcNAc-1-P uridyltransferase are suppressed. This microorganism contains additional genetic modifications that increase phosphoglucoisomerase, glutamine synthetase and glucose 6-P dehydrogenase activities and inactivate phosphofructokinase and genes encoding the enzymes responsible for glycogen synthesis to control the flux of metabolites. Because glucose is the major substrate for GlcNAc production, fructose is supplemented to maintain the energy supply for bacterial proliferation. In this process, a high concentration (exceeding 120 g/L) of GlcNAc was obtained after fermentation for 60 h and is, thus, the most efficient process by far.

## 3. Applications of GlcNAc

GlcNAc belongs to a large class of amino sugars that serve a number of functions and are located throughout the human system. For example, GlcNAc and its analogues, such as chitin and chitosan, are known as the carbon and/or nitrogen sources for creatures. In fact, they also could be used as a biomass. It has been shown that *Rhodotorula glutinis* is able to convert GlcNAc to biofuel [92,93]. The bioethanol could be yielded with the GlcNAc from chitin waste as a C6 carbon source [94].

GlcNAc is also a component of glycoproteins, proteoglycans, GAGs and other connective tissue building blocks [42]. GAGs and glycoproteins act as substrate materials for tissue repair and anti-inflammatory reactions [95]. When GlcNAc is present in the medium of pig gastric mucosal cells, it is incorporated into high molecular weight glycoproteins [96]. Additionally, after 168 h of oral administration of GlcNAc, about 25% of GlcNAc accumulates in tested animals [97]. This finding indicates that GlcNAc is an important component of biomacromolecular synthesis in the body.

Certain diseases appear to be related to abnormalities in the formation and utilization of amino sugars. Cell membranes, intercellular fluids and cell regeneration could all be affected by GlcNAc. In recent years, GlcNAc has been discovered to be a valuable pharmacological agent in the treatment of a wide variety of ailments.

### 3.1. Safety of GlcNAc

A large dose of GlcNAc (20 g) given intravenously to human volunteers results in neither toxicity nor alteration of blood glucose concentration [42,43]. A lack of insulin resistance was also found after oral administration of GlcNAc, even at a high dose. Systematic toxicological tests have been performed in animals, including the acute toxicity test, Ames test, micronucleus test of mouse bone marrow cells, abnormality test of mouse sperm, aberration test of mouse testis chromosomes, chronic lethal test, sub-chronic toxicity test (for 90 days) and traditional deformity-inducing test [98,99]. These experiments indicate that GlcNAc is a valuable compound with high safety in invaded, oral and topical usage. GlcNAc has been widely utilized as a nutritional supplement for therapeutic usage. It is classified as described in the following sections.

### 3.2. GlcNAc is used to treat joint damage

Articular cartilage is a matrix of proteoglycans, chondrocytes and collagens. It absorbs shock from mechanical forces and provides a smooth surface so that bone ends may glide easily across one another. Chondrocytes are known to contribute to the synovial fluid, which bathes the articular cartilage. Lack of proteoglycan precursors and synovial fluid leads to defects in the structure and function of skeletal joints and osteoarthritis occurs. The incidence increases with age and is found in most people over the age of 65 [100,101].

Many clinical trials have been performed to treat patients with joint disorders, including arthritic diseases, osteoarthritis, rheumatoid arthritis, cartilage damage, joint injury and degenerative joint disease (DJD). Preparations containing GlcNAc are delivered by parenteral, oral, transmucosal and topical administration. The results from these deliveries indicate that GlcNAc significantly enhances the prevention of joint damage [102–107]. Moreover, GlcNAc also inhibits elastase activity and superoxide release from human polymorphonuclear leukocytes [108,109].

### 3.3. GlcNAc is a potential candidate to treat inflammatory bowel disease (IBD)

Mucous membranes of the entire digestive tract are composed of epithelial cells, which have a high rate of turnover. The synthesis of glycoproteins and GAGs by the mucosa is considered to provide protection for the lining of bowel. IBD is a generic diagnosis that encompasses a number of bowel diseases, including ulceracolitis, chronic proctitis and Crohn’s disease. IBD can involve the inflammatory disruption of vascular and matrix GAGs, and tumor necrosis factor-α appears to play an important role in inflammation [110–112]. The loss of GAGs from the intestinal wall results in fibrosis of the bowel. Tissue defects in the digestive tract result in food intolerance, in which food allergens are absorbed by the gastrointestinal tract, followed by the development of psoriasis [113,114].

GlcNAc is capable of enhancing the release of acid mucopolysaccharides by fibroblasts and restoring the formation of the protective structure of the gastrointestinal tract [115]. In addition, GlcNAc increases the elasticity of perivascular tissue, resulting in an increase in arterio-capillary blood flow. GlcNAc acts as a cytoprotective agent for restoring the integrity and normal function of the mucous membrane in humans [116,117]. A pilot clinical trial has been performed to identify the therapeutic effect of GlcNAc on IBD. Children with symptomatic Crohn’s disease showed clear improvement after either oral or rectal administration of GlcNAc [118]. Therefore, GlcNAc shows promise as an inexpensive and non-toxic treatment for chronic IBD. Recently, a phase III clinical trial has just been finished [43].

### 3.4. Application of GlcNAc in cosmetics

Human skin contains stratum corneum and dermis to protect the body from harsh environmental conditions, such as dryness and UV irradiation. Stratum corneum plays a key role in maintaining the moisture and firmness of the skin. It consists of flattened dead cells, corneocytes and a complex of lipid matrix and natural moisture factors [119]. Dermis, the inner layer of the skin, is composed of collagen, elastin and GAGs, which are created by fibroblasts. Collagen is responsible for the resilience, strength, durability, elasticity, smoothness and plump appearance of healthy skin. Mucopolysaccharides, such as HA and proteoglycans, have a high water retention capacity and are important components in maintaining skin moisture.

When the amount of mucopolysaccharides decreases with age, the water retention and resilience of the skin decreases, and thereby causes rough skin and fine wrinkles. Cosmetics, commercially available topical preparations have been designed to increase the moisture content of the skin. Most cosmetics provide only a short-term replenishment of moisture-providing ingredients. Skin care ingredients that are derived from natural substances with high safety ratings are regarded as more beneficial.

HA, which is produced mainly by fibroblasts and keratinocytes, is a well known ingredient that holds water in the stratum corneum and dermis [120]. HA content is reported to decline with age, which may contribute to wrinkle formation and the decrease in elasticity of the skin [121]. HA is suggested to be a valuable ingredient in cosmetics. However, HA absorb inefficiently by either topical or oral administration due to its high molecular weight. Addition of GlcNAc, which is the building unit of HA, to cultured keratinocytes results in a dose-dependent increase in the production of HA [122], but has no effect on HA production of skin fibroblasts. Instead, GlcNAc enhances the proliferation and collagen expression of fibroblasts [123].

Topical administration of GlcNAc is used to improve skin quality. GlcNAc has been shown to be a good skin penetrant based on *in vitro* Franz cell testing. This result is consistent with the joint pain relieving effect of GlcNAc by topical administration [106]. In addition to its moisturizing effects, GlcNAc has been found to reduce the appearance of facial hyperpigmentation in an 8-week, double-blind clinical trial [124]. Using *in vitro* genomic experiments, the mechanism by which GlcNAc reduces melanin production has been found to involve the up-regulation of several genes, such as epidermal turnover genes and antioxidant-related genes, and the down-regulation of the cytoskeleton genes involved in melanosome transport. These changes are believed to be associated with pigment reduction [125]. Due to the versatile functions of GlcNAc, it is considered a valuable ingredient in cosmetics for improving skin wrinkles and color [126,127]. Because GlcNAc promotes the proliferation of keratinocytes and fibroblasts and increases the production of HA in the skin, it has also been successfully used to heal wounds [128,129].

### 3.5. GlcNAc is used as a substrate in sialic acid production

*N*-Acetylneuraminic acid (Neu5Ac) is the most common sialic acid and exists as >40 structural derivatives in mammalian and avian species [130]. Sialic acids are found at the distal ends of cell surface glycoconjugates and are major determinants of cellular recognition [131], serving as receptors for influenza viruses [131–133].

Infection of host cells by influenza viruses begins with the binding of hemagglutinin on the virus to the end of the sugar chain on the host cell. The infiltrating virus continuously proliferates in the cell. At the moment when the virus is to be released from the cell, the neuraminidase located on the surface of the virus hydrolyzes the binding site on the superficial layer of the host cell, and the freed virus binds to other non-infected cells. Novel pharmaceutical agents that are chemically synthesized from Neu5Ac to produce neuraminidase inhibitors are used to treat influenza infections. Indeed, Zanamivir, the guanidyl derivative of Neu5Ac, has been approved by several governments as an effective remedy for the treatment of the flu [132,133].

Because the Neu5Ac content in natural products is too low for the isolation of Neu5Ac with sufficient recovery and purity, novel processes had to be developed for the industrial production of this compound. Enzymatic synthesis of Neu5Ac from *N*-acetylmannosamine (ManNAc) and pyruvate using Neu5Ac aldolase as a catalyst has been reported. However, because ManNAc is very expensive, methods for the preparation of ManNAc from inexpensive GlcNAc, using chemical epimerization or enzymatic conversion by GlcNAc 2-epimerase, have been developed [134]. The simple and large-scale production of Neu5Ac using a two-step reaction was performed by Maru *et al*., in which 29 kg of Neu5Ac was obtained from 27 kg of GlcNAc, using recombinant GlcNAc 2-epimerase and Neu5Ac lyase as catalysts [135]. Lee *et al*. (2007) also developed a method to produce Neu5Ac using recombinant whole cells that express GlcNAc 2-epimerase and recombinant Neu5Ac lyase separately [136]. In summary, GlcNAc is an important substrate for the production of sialic acids.

### 3.6. Other applications of GlcNAc

A novel GlcNAc application has been developed by Xu *et al.* Because GlcNAc is a pure compound with a high level of safety, it is an appropriate candidate for multiple applications, especially for drug development. To test the special functions of GlcNAc, a novel bio-wave model was established with the use of *Proteus mirabilis*. In this model, the swarm colonies of *P. mirabilis* extend outward, and concentric rings are formed on the agar within 3 h as a result of the decay of metabolites [137]. In the presence of GlcNAc, many fine waves are formed in each ring, in addition to the typical concentric rings. Thus, GlcNAc was shown to portray a finer bio-wave characteristic than other compounds. This wave-promoting function may explain the special applications of GlcNAc described in Table 1. A series of cytological tests, animal tests and clinical trials have been performed to confirm the special functions of GlcNAc. Some of the successful cases have been filed as patents and are being utilized in the development of new drugs.

## 4. Conclusions

GlcNAc is prepared using chitin as a substrate by chemical, enzymatic and biotransformation methods. New methods using glucose as a substrate can also be applied to obtain GlcNAc using genetically modified microorganisms. Finally, the versatile functions of GlcNAc promote its use as a novel candidate for drug development.

## Figures and Tables

**Table 1:** Applications of GlcNAc derived from the bio-wave model

Function	Object	Trial	Reference
Disease Treatment	cancer and metastasis	animal, human	[138]
	autoimmune reactions	cell, animal, human	[139]
	urogenital tract infection	cell, human	[140]
	viral or bacterial infection	cell, animal	[141]
	sexual disorder	human	[142]
	non-specific inflammation	animal, human	[143]
	perianal disease	cell, animal, human	[144]
	intestinal disease	human	[145]
	cervical erosion	bacteria, human	[146]
	respiratory tract disease	human	[147]

Disease Prevention	toxicosis of drugs or chemicals	cell, animal	[148]
	antibiotics	bacteria	[149]
	side effects of radiotherapy and chemotherapy	human	[150]
	cardio-cerebrovascular anoxemia	animal	[151]
	motion sickness	human	[152]

Dermatology	modulation of microorganisms on mucus membrane	animal, human	[153]
	skin sanitary article preparation	human	[154]
	microecological balance of mucosa	bacteria, human	[155]

Food supplement	additive in milk	animal, human	[156]
	additive in beer	animal, human	[157]
	additive in wine	animal, human	[158]
